# Identification and characterization of auxiliary proteins encoded by the STLV-3 retrovirus pX region

**DOI:** 10.1186/1742-4690-8-S1-A133

**Published:** 2011-06-06

**Authors:** Jocelyn Turpin, Theresa Nga Ling Ko, Julien Villaudy, Amandine Galioot, Antoine Gessain, Louis Gazzolo, Madeleine Duc Dodon, Renaud Mahieux

**Affiliations:** 1 Virologie Humaine, INSERM-U758, Lyon, France; 2 Ecole Normale Supérieure de Lyon, Lyon, France; 3 IFR 128 BioSciences Lyon-Gerland, Lyon, 69364,Cedex 07, France; 4 Unité d’Epidémiologie et Physiopathologie des Virus Oncogènes, CNRS URA 3015, Institut Pasteur, Paris, 75015, France

## 

The PTLV-3 group includes simian viruses (STLV-3) and the recently identified human viruses (HTLV-3). These viruses display a high percentage (>95%) of sequence identity. Recent studies have shown that the auxiliary proteins of complex retroviruses such as HIV and HTLV-1 are playing an important role in the viral life cycle in vivo. However, it has not been determined yet whether the genome of the Primate T-cell Lymphotropic viruses, type 3 encodes such proteins.

To uncover the potential presence of auxiliary proteins, we first extracted RNA from cells either infected with STLV-3 or transfected with a STLV-3 infectious molecular clone. RT-PCR experiments using primers specific of the pX region allowed the amplification of two different doubly spliced mRNAs, one encoding a putative 63 amino-acid protein and another one encoding a putative 79 amino-acid protein. Based on the molecular weight prediction, we named these proteins p8 and p9, respectively. The p8 sequence is present in 90% of all HTLV-3 and STLV-3 strains. The N-Ter 21 amino acid sequence is shared with the corresponding Rex3 sequence. This sequence was found to be homologous to that of Rex1, which contains the nucleolar localization signal, and the RNA binding domain. Interestingly, after transfection of a p8 expression vector, we observed that the protein localized within the nucleolus. We are proceeding to the characterization of p8 subdomains as well as to the functional analyses of the p8 functions. These experiments will allow us to determine whether p8 represent the counterpart of an HTLV-1 auxiliary protein.

